# Peripheral complement proteins in schizophrenia: A systematic review and meta-analysis of serological studies

**DOI:** 10.1016/j.schres.2020.05.036

**Published:** 2020-08

**Authors:** David Mongan, Sophie Sabherwal, Subash Raj Susai, Melanie Föcking, Mary Cannon, David R. Cotter

**Affiliations:** Department of Psychiatry, Royal College of Surgeons in Ireland, Dublin, Ireland

**Keywords:** C3, complement component 3, C4, complement component 4, CHR, clinical high-risk for psychosis, ELISA, enzyme-linked immunosorbent assay, FEP, first episode psychosis, MBL, mannose-binding lectin, Complement, Schizophrenia, Psychosis, Systematic review, Serum, Plasma

## Abstract

**Background:**

There is renewed focus on the complement system in the pathogenesis of schizophrenia. In addition to providing aetiological insights, consistently dysregulated complement proteins in serum or plasma may have clinical utility as biomarkers.

**Methods:**

We performed a systematic literature review searching PubMed, Embase and PsycINFO for studies measuring complement system activity or complement protein concentrations in serum or plasma from patients with schizophrenia compared to controls. Random-effects meta-analyses were performed to calculate pooled effect estimates (Hedges' g standardised mean difference [SMD]) for complement proteins whose concentrations were measured in three or more studies. The review was pre-registered on the PROSPERO database (CRD42018109012).

**Results:**

Database searching identified 1146 records. Fifty-eight full-text articles were assessed for eligibility and 24 studies included. Seven studies measured complement system activity. Activity of the classical pathway did not differ between cases and controls in four of six studies, and conflicting results were noted in two studies of alternative pathway activity. Twenty studies quantified complement protein concentrations of which complement components 3 (C3) and 4 (C4) were measured in more than three studies. Meta-analyses showed no evidence of significant differences between cases and controls for 11 studies of C3 (SMD 0.04, 95% confidence interval [CI] -0.29–0.36) and 10 studies of C4 (SMD 0.10, 95% CI -0.21–0.41).

**Conclusions:**

Serological studies provide mixed evidence regarding dysregulation of the complement system in schizophrenia. Larger studies of a longitudinal nature, focusing on early phenotypes, could provide further insights regarding the potential role of the complement system in psychotic disorders.

## 1 Introduction

### 1.1 Rationale

Schizophrenia is a severe mental disorder, among the most disabling conditions worldwide (Collaborators, 2017). While the precise pathogenesis remains unknown, hypotheses for involvement of the immune system and inflammation have developed from several lines of enquiry. For example, infants born to mothers exposed to the influenza virus in pregnancy are at increased risk of developing schizophrenia later in life (Limosin et al., 2003). Epidemiological studies suggest associations between several autoimmune diseases and psychosis (Benros et al., 2014; Cullen et al., 2019). Several studies have reported raised serological markers of inflammation in patients with schizophrenia compared to controls. In their meta-analysis, Miller et al. identified multiple inflammatory cytokines consistently raised in patients with schizophrenia compared to controls (Miller et al., 2011). In a subsequent meta-analysis of patients with first-episode psychosis (FEP), several cytokines were again found to be raised in comparison to controls (Upthegrove et al., 2014).

Recently there has been renewed focus in schizophrenia research on the complement system, an important component of innate immunity named for its ‘complementary’ role in conjunction with adaptive immune responses. Holers (2014) provides a detailed discussion of the components and functions of the complement system. To summarise, the complement system is a complex network of approximately 60 interacting proteins that, upon activation, augments immune and inflammatory responses through opsonisation of antigens, chemotaxis and, ultimately, formation of a membrane attack complex (C5b-9) capable of rupturing invading bacterial cells (Mayilyan et al., 2008b; Nimgaonkar et al., 2017). Activation of the system occurs via three main routes known as the classical, alternative and mannose-binding lectin (MBL) pathways. While each pathway is initiated by different molecular mechanisms, all three lead to activation of the central complement protein known as complement component 3 (C3). C3 and its activation products promote opsonisation and chemotaxis as well as contributing to formation of the membrane attack complex. A range of control proteins, such as complement factor H, complement factor I and C4b binding protein, regulate activity of the system (Zipfel and Skerka, 2009).

The complement system serves vital immune functions in health by augmenting the clearance of micro-organisms and necrotic cells. However, dysregulation or inappropriate activation in the presence of self-antigens can contribute to disease states, as is associated with disorders such as systemic lupus erythematosus (Popescu and Kao, 2011), multiple sclerosis (Ingram et al., 2009; Michailidou et al., 2016) and Alzheimer's disease (Hong et al., 2016a; VanItallie, 2017). Genome-wide association studies have associated schizophrenia susceptibility with polygenic variation in the major histocompatibility complex (Consortium, 2014), a region containing a set of genes that code for multiple immune-related proteins, including several complement components. Sekar et al. (2016) showed that this association was partly related to allelic variation of the complement component 4 (C4) gene. The same authors also found that C4 RNA expression was increased in post-mortem brain tissue from patients with schizophrenia compared to controls. These findings have spurred interest regarding the role of the complement system in schizophrenia. In the brain, complement proteins are thought to play a role in tagging synapses for elimination by microglia (Stephan et al., 2012) which could represent an underlying biological mechanism for the observations of reduced synaptic density in schizophrenia (Osimo et al., 2018).

Aside from providing aetiopathogenic insights, if peripheral complement proteins are consistently dysregulated in schizophrenia then they may have clinical utility as biomarkers. Investigators have generally used two broad categories of assays to study this area: activity-based assays and concentration-based assays. Activity-based assays typically assess haemolytic activity by measuring the ability of serum to lyse sheep erythrocytes sensitised with rabbit immunoglobulin M for classical pathway activity, or unsensitised rabbit erythrocytes for alternative pathway activity under conditions that block functioning of the classical pathway (Costabile, 2010; Oppermann and Wurzner, 2010). Assays are also available that measure functional (rather than haemolytic) activity, such as using enzyme-linked immunosorbent assay (ELISA) techniques to measure the formation of membrane attack complexes after complement activation (Seelen et al., 2005). Concentration-based assays, rather than providing a measure of activity, quantify the concentration of individual complement proteins in the blood. This may be performed using several methods including ELISA, nephelometry, radial immunodiffusion and other techniques (Kirschfink and Mollnes, 2003; Nilsson and Ekdahl, 2012).

### 1.2 Objective

The objective of this review was to systematically synthesise and evaluate the available evidence from serological studies to answer the question: in patients with schizophrenia, are peripheral complement protein concentrations or complement activity consistently altered in comparison to controls without the disorder?

## 2 Methods

### 2.1 Protocol and registration

The protocol for this review is registered on the PROSPERO database (https://www.crd.york.ac.uk/prospero/; registration number CRD42018109012).

### 2.2 Eligibility criteria

Inclusion criteria for studies were: case-control or cohort studies; comparing serum or plasma complement protein concentrations or activity in patients diagnosed with schizophrenia to controls without schizophrenia; published in peer-reviewed journals; available in English. Review articles that did not present original data were included in the primary search so that their reference lists could be screened for potential additional records.

Exclusion criteria for studies were: genetic or RNA expression studies; non-human studies; no healthy control comparison group; poster or conference abstracts; studies measuring the same proteins in a previously-presented sample; studies focusing exclusively on psychiatric disorders other than schizophrenia (for example depression or bipolar disorder). We chose to focus on studies that used targeted approaches measuring complement proteins or their activity. Thus, studies that relied upon proteome-wide or discovery-based approaches (such as ‘shotgun’ proteomics by mass spectrometry) were not included. Evidence derived from such methods in schizophrenia (Sabherwal et al., 2016) and other severe mental disorders (Comes et al., 2018) has been synthesised in recent systematic reviews.

### 2.3 Information sources and search strategy

We searched PubMed, Embase and PsycINFO from inception to current date using search strategies optimised for each database. The search strategy was developed and performed in collaboration with an information specialist (the full list of search terms for each database is included in supplementary appendix A). The search was last performed on 17th June 2019. Reference lists of included studies were also examined for further potential records.

### 2.4 Study selection

Titles and abstracts were screened independently by two authors (DM and SS). Full-text articles were obtained for the remaining records, which were assessed for inclusion according to the eligibility criteria above. Differences were resolved by discussion and involvement of a third author where necessary.

### 2.5 Data collection process

For each included study, data were extracted by two authors independently (DM and SS) using a custom-developed and pre-piloted data extraction form. Key data items extracted included country of origin; case definition and method of diagnosis; hospitalisation status of cases; number of cases and controls; type of assay used; complement pathways or proteins measured and their mean values and standard deviations (or other summary measures where appropriate) in cases and controls. Where data were unavailable or unclear, we attempted to contact the study authors. Where data were presented in graphical form only (and raw data unavailable from authors), data extraction software was used to estimate the numerical values (WebPlotDigitizer version 4.2; https://automeris.io/WebPlotDigitizer/).

### 2.6 Risk of bias assessment

Risk of bias was assessed at the study level using the Newcastle-Ottawa quality assessment scale (Wells et al., 2013). This measure consists of three subscales used to assess study quality, with a maximum score of nine points: four points for selection of cases and controls, two points for comparability between cases and controls and three points for determination of exposure.

### 2.7 Meta-analyses

We proposed to perform meta-analyses for complement proteins measured using concentration-based assays in at least three studies. Among all proteins measured across the included studies, C3 and C4 met this criterion. Sample size, mean and standard deviation values in cases and controls for concentrations of these proteins were recorded. Where studies presented the standard error of the mean, we converted this to the standard deviation. Where studies presented the median and interquartile range, we attempted to contact authors for the mean and standard deviation values.

Statistical analyses were performed using Stata version 15 (StataCorp). Predicting significant between-study heterogeneity, we used random-effects models to calculate pooled effect size estimates (Hedges' *g* standardised mean difference [SMD]). Statistical heterogeneity was assessed using the *I*^2^ statistic. For all analyses, the threshold for statistical significance was *p* = .05.

#### 2.7.1 Publication bias

Evidence for publication bias was assessed via inspection of funnel plots. Evidence for small-study effects was assessed using Egger's test (Egger et al., 1997).

#### 2.7.2 Meta-regression analyses

Post-hoc, we performed univariate meta-regression analyses to explore heterogeneity by evaluating the effects of three study characteristics upon the effect estimates: medication status at time of blood sampling (concurrent use of antipsychotics vs. no concurrent use), hospitalisation status of cases (inpatient vs. outpatient) and assay methodology (ELISA vs. radial immunodiffusion or other; radial immunodiffusion vs. ELISA or other; and other vs. ELISA or radial immunodiffusion).

## 3 Results

### 3.1 Study selection

**Fig. 1:**
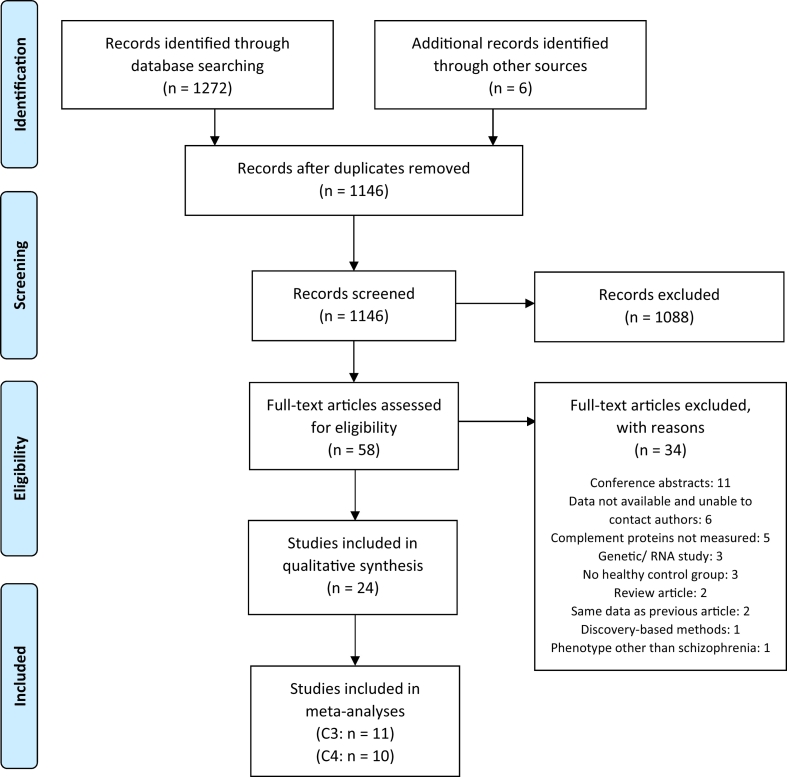
PRISMA flow chart showing search strategy and results. C3: complement component 3; C4: complement component 4.

Fig. 1 shows the results of the search strategy and study selection process, which was performed and is reported in accordance with Preferred Reporting Items for Systematic Reviews and Meta-Analyses (PRISMA) guidelines (Moher et al., 2009). Database searching identified 1272 records (462 from PubMed, 373 from Embase and 437 from PsycINFO) and 6 records were identified from reference screening. Following removal of duplicates, 1146 title and abstract records were screened of which 1088 were excluded. Full-text articles were obtained for the remaining 58 records, which were assessed for eligibility according to the inclusion and exclusion criteria. Thirty-four articles were excluded for multiple reasons (detailed in Fig. 1) resulting in 24 included studies (Ali et al., 2017; Boyajyan et al., 2010; Cazzullo et al., 1998; Foldager et al., 2012; Fontana et al., 1980; Hakobyan et al., 2005; Hong et al., 2016b; Idonije et al., 2012; Ji et al., 2019; Kucharska-Mazur et al., 2014; Laskaris et al., 2018; Li et al., 2016; Li et al., 2012; Maes et al., 1997; Mayilyan et al., 2006; Mayilyan et al., 2008a; Ramsey et al., 2013; Santos Soria et al., 2012; Sasaki et al., 1994; Schwarz et al., 2012; Spivak et al., 1993; Spivak et al., 1989; Walss-Bass et al., 2019; Wong et al., 1996).

From: Moher et al. (2009). *P*referred *R*eporting *I*tems for *S*ystematic Reviews and *M*eta-*A*nalyses: The PRISMA Statement. PLoS Med 6(7): e1000097. doi:https://doi.org/10.1371/journal.pmed1000097

### 3.2 Study characteristics

Characteristics of studies included in this review are presented in Table 1 (studies using activity-based assays) and Table 2 (studies using concentration-based assays). Unadjusted values for assay results are presented unless otherwise stated.

**Table 1:** Summary of included studies using activity-based assays.

Study	Country	Hospitalisation status of cases	Diagnostic criteria	Medication status	Sample size	Measure	Sample type	Assay	Main findings	Notes
Boyajyan et al., 2010	Armenia	Inpatient	ICD-10	Medicated and drug-free	36 medicated cases, 25 drug-free cases vs. 26 controls	Alternative pathway activity, C3 activity	Serum	Haemolytic activity	Mean alternative pathway activity in medicated cases (228.3 AH50 units/ml, *p* = .001) and drug-free SZ (155.6 AH50 units/ml, *p* = .001) significantly ↑ vs. controls (93.7 AH50 units/ml) Mean C3 activity in medicated cases (294.1 C3H50 units/ml, *p* = .001) and drug-free SZ (138.5 C3H50 units/ml, *p* = .002) significantly ↑ vs. controls (99.5 C3H50 units/ml)	All cases had multiple-episode SZ of paranoid subtype No difference between smokers and non-smokers or males and females
Hakobyan et al., 2005	Armenia	Inpatient	ICD-10	Medicated and drug-free (for mean of 10 weeks)	24 cases (13 drug-free) vs. 28 controls	Classical pathway activity and activities of C1, C2, C3 and C4	Serum	Haemolytic activity	Mean classical pathway activity not significantly different between cases (39.24 CH50 units) vs. controls (37.31 CH50 units) Mean C1 activity significantly ↑ in cases (39.10 CH50 units) vs. controls (22.90 CH50 units), *p* < .002 Mean C2 activity significantly ↓ in cases (3.52 CH50 units) vs. controls (6.82 CH50 units), *p* < .001 Mean C3 activity significantly ↑ in cases (204.83 CH50 units) vs. controls (101.88 CH50 units), *p* < .004 Mean C4 activity significantly ↑ in cases (944.57 CH50 units) vs. controls (686.37), *p* < .001	Cases were in acute relapse of multiple-episode paranoid SZ and had first- or second-degree family history of SZ No difference between smokers and non-smokers or males and females Only mean C3 activity significantly differed between medicated (289.53 CH50 units) and drug-free cases (133.16 CH50 units), *p* = .001
Li et al., 2012	China	Outpatient	DSM-IV	Drug-free	47 cases (29 antipsychotic naïve, 18 drug-free for 8 weeks) vs. 53 controls	Classical pathway, alternative pathway and MBL pathway activity	Serum	Qualitative ELISA	Alternative pathway activity significantly ↓ in drug-free cases (75%) vs. medicated cases (82%), p = .001; and drug-free cases vs. controls (86%), p = .001 No evidence of significant difference between drug-free or drug-treated cases and controls for classical or MBL pathways	
Mayilyan et al., 2006	Armenia	Outpatient	DSM-IV	Medicated and drug-free (>3 months)	45 cases (9 drug-free) vs. 29 controls	Classical pathway activity (CH50), C4 activity, MBL-bound MASP1 activity, MBL-bound MASP2 activity	Serum	Haemolytic activity	Median total complement activity significantly ↑ in cases (1334 units/ml) vs. controls (1087 units/ml), p < .02 Median C4 activity significantly ↑ in cases (143,000 U/ml) vs. controls (109,000 U/ml), *p* < .02 Mean MBL-bound MASP-2 activity significantly ↑ in cases (7.26 U/ml) vs. controls (5.66 U/ml), *p* < .01 No significant difference in MBL-bound MASP1 activity between cases and controls	All cases diagnosed with paranoid subtype and had first- or second-degree family history of SZ
Sasaki et al., 1994	Japan	Inpatient	DSM-III-R	Medicated and drug-free	14 cases (3 drug-free) vs. 20 controls	Classical pathway activity (CH50)	Serum	Haemolytic activity	Mean CH50 not significantly different between cases (36.7 units/ml) and controls (36.3 units/ml)	Cases had chronic SZ, in acute relapse
Spivak et al., 1989	Israel	Inpatient	DSM-III	Drug-free (≥3 months)	20 cases vs. 18 controls	Classical pathway activity (CH100)	Serum	Haemolytic activity	Mean CH100 not significantly different between cases (69.1 units/ml) and controls (75.9 units/ml)	Controls recruited from medical staff
Spivak et al., 1993	Israel	Inpatient	DSM-III-R	Drug-naïve, drug-free (≥3 months) and medicated	91 cases (20 drug-naïve, 37 drug-free, 34 medicated) vs. 37 controls	Classical pathway activity (CH100)	Serum	Haemolytic activity	Mean CH100 significantly ↓ in cases (65 units/ml) vs. controls (85 units/ml), *p* = .007 No differences between cases vs. controls when stratified by medication status	Cases had chronic SZ Controls recruited from medical staff

SZ: schizophrenia; ELISA: Enzyme-linked immunosorbent assay; ICD: International Classification of Diseases; DSM: Diagnostic and Statistical Manual; MBL: mannose-binding lectin; MASP: mannose-binding lectin serine protease.

**Table 2:** Summary of included studies using concentration-based assays.

Study	Country	Hospitalisation status of cases	Diagnostic criteria	Medication status of cases	Sample size	Complement proteins	Sample type	Assay	Main findings	Notes
Ali et al., 2017	Egypt	Outpatient	DSM-IV	Drug-free	44 cases vs. 50 controls	C3, C4	Serum	ADVIA Chemistry XPT System	Mean C3 in cases (169.1 mg/dl) significantly ↑ vs. controls (133.5 mg/dl), *p* < .001 Mean C4 in cases (33.9 mg/dl) not significantly different vs. controls (31.3 mg/dl)	C3 and C4 were not significantly correlated with PANSS score
Cazzullo et al., 1998	Italy	Not known	DSM-III-R	Medicated	29 cases vs. 20 controls	C3, C4	Serum	Nephelometry	Median C3 in cases (155 mg/dl) not significantly different vs. controls (134 mg/dl) Median C4 in cases (33 mg/dl) not significantly different vs. controls (28 mg/dl)	Cases were treatment-resistant with active SZ symptoms C4 values estimated from graph
Foldager et al., 2012	Denmark	Inpatient and outpatient	ICD-10, DSM-IV	Medicated	100 cases vs. 350 controls	MBL, MASP-2	Serum	Time-resolved immuno-fluorometric assay	Median MBL in cases (584 ng/ml) not significantly different vs. controls (578 ng/ml); significantly higher in cases when adjusted for MBL2 haplotypes Median MASP-2 in cases (425 ng/ml) not significantly different vs. controls (417 ng/ml)	Samples for cases obtained from previous genetic studies and acutely psychotic at time of sampling Controls were blood donors, not psychiatrically screened
Fontana et al., 1980	Switzerland	Inpatient	ICD	Medicated	48 cases vs. 100 controls	C3, C4, C1-inhibitor	Serum	C3 by radial immunodiffusion C4 and C1-inhibitor by immuno-electrophoresis	Mean C3 not significantly different between cases (130.2 mg/ml) vs. controls (150.0 mg/ml) Mean C4 not significantly different between cases (16.7 mg/ml) vs. controls (18.6 m/ml) Mean C1-inhibitor not significantly different between cases (17.0 mg/ml) vs. controls (21.5 mg/ml)	Controls were blood donors
Hong et al., 2016b	China	Inpatient	ICD-10	Drug-naïve and drug-free (≥4 weeks)	41 cases vs. 33 controls	C3	Plasma	ELISA	Mean C3 not significantly different between cases (161.19 μg/ml) vs. controls (164.20 μg/ml)	Controls recruited from medical staff
Idonije et al., 2012	Nigeria	Inpatient	DSM-IV	Medicated and drug-naïve	20 chronic medicated cases, 15 first episode drug-naïve cases vs. 20 controls	C1q, C3c, C4, C5, C1 inhibitor, C3 activator	Serum	Immunoplates	Mean C1q significantly ↓ in chronic cases (3.61 g/l) vs. controls (9.40 g/l), *p* < .001; significantly ↓ in first episode cases (3.09 g/l) vs. controls, *p* < .001; no significant difference between chronic and first episode cases Mean C3c not significantly different between chronic cases (1.30 g/l) vs. controls (1.41 g/l); significantly ↓ in first episode cases (0.68 g/l, p < .001) vs. controls; significantly ↑ in chronic cases vs. first episode cases (p < .001) Mean C4 not significantly different between chronic cases (0.17 g/l) vs. controls (0.26 g/l) or first episode cases (0.23 g/l) vs. controls; no significant difference between chronic cases vs. first episode cases Mean C5 not significantly different between chronic cases (1.55 g/l) vs. controls (1.50 g/l) or first episode cases (1.20) vs. controls; significantly ↑ in chronic cases vs. first episode cases, p 0.04 Mean C1 inhibitor not significantly different between chronic cases (1.21 g/l) vs. controls (0.53 g/l) or first episode cases (0.49 g/l) vs. controls or chronic cases vs. first episode cases Mean C3 activator not significantly different between chronic cases (0.75 g/l) vs. controls (0.89 g/l); or first episode cases (0.66 g/l) vs. controls; or chronic cases vs. first episode cases	
Ji et al., 2019	China	Inpatient	ICD-10	Not known	40 cases vs. 40 controls	C4	Serum	ELISA	Mean C4 significantly ↓ in cases (154.2 μg/ml) vs. controls (216.2 μg/ml), p < .001	Cases had first episode SZ
Kucharska-Mazur et al., 2014	Poland	Not known	ICD-10	Drug-naïve	22 cases vs. 35 controls	C3a, C5a, C5b-9	Plasma	ELISA	Mean C3a significantly ↓ in cases (494.33 ng/ml) vs. controls (591.13 ng/ml), p 0.03 Mean C5a not significantly different between cases (52.94 ng/ml) and controls (53.24 ng/ml) Mean C5b-9 not significantly different between cases (355.03 ng/ml) and controls (220.73 ng/ml)	Cases had first episode SZ (22 of 28 recruited patients with first episode psychosis)
Laskaris et al., 2018	Australia	Outpatient	DSM-IV	Medicated	50 cases vs. 54 controls	C1q, C3 and C4	Serum	ELISA multiplex	Mean C1q not significantly different between in cases (48.9 μg/ml) vs. controls (45.8 μg/ml) Mean C3 not significantly different between cases (16.9 μg/ml) vs. controls (17.1 μg/ml) Mean C4 significantly ↑ in cases (292.2 μg/ml) vs. controls (252.0 μg/ml), *p* = .04	Cases had chronic SZ Values adjusted for BMI
Li et al., 2016	China	Inpatient	ICD-10	Drug-free (≥ 4 weeks)	40 cases vs. 40 controls	C3	Plasma	ELISA	Median C3 significantly ↓ in cases (118,742.49 ng/ml) vs. controls (160,853.17 ng/ml), p = .04	Controls recruited from medical staff C3 negatively correlated with PANSS score (r − 0.37, *p* = .03)
Maes et al., 1997	USA	Inpatient	DSM-III-R	Drug-free (≥1 week) and medicated	27 cases (17 drug-free, 10 medicated) vs. 21 controls	C3C, C4	Plasma	Nephelometry	Mean C3c significantly ↑ in drug-free cases (92 mg/dl) vs. controls (74 mg/dl), p = .03; not significantly different between medicated cases (89 mg/dl) vs. controls Mean C4 significantly ↑ in drug-free cases (41 mg/dl) vs. controls (29 mg/dl), *p* = .02; not significantly different between medicated cases (37 mg/dl) vs. controls	Values adjusted for age and gender
Mayilyan et al., 2006	Armenia	Outpatient	DSM-IV	Drug-free (>3 months) and medicated	45 cases (9 drug-free) vs. 29 controls	MBL	Serum	ELISA	Mean MBL serum concentration not significantly different between cases (1.74 μg/ml) vs. controls (2.55 μg/ml)	Cases had chronic SZ in remission and had positive family history of SZ
Mayilyan et al., 2008a	Armenia	Outpatient	ICD-10, DSM-IV	Drug-free (≥ 2 months) and medicated	45 cases (9 drug-free) vs. 51 controls	C4B	Serum	ELISA	Median C4B significantly ↓ in cases (159.1 mg/l) vs. controls (180.0 mg/l), p < .01	Cases had chronic SZ in remission
Ramsey et al., 2013	Germany	Not known	DSM-IV	Drug-naïve	133 cases vs. 133 controls	C3	Serum	ELISA multiplex	C3 significantly ↑ in cases (ratio cases:controls 1.07), p = .002 (*p* = .013 corrected for multiple comparisons)	Cases had first episode SZ
Santos Soria et al., 2012	Brazil	Outpatient	DSM-IV	Medicated	53 cases vs. 80 controls	C3, C4	Serum	Immuno-turbidimetry	Mean C3 significantly ↑ in cases (190.3 mg/dl) vs. controls (162.6 mg/dl), p < .01 Mean C4 not significantly different between cases (40.3 mg/dl) vs. controls (38.4 mg/dl)	Cases had chronic SZ in remission
Schwarz et al., 2012	Germany, Holland, UK	Inpatient	DSM-IV	Drug-naïve	71 cases vs. 59 controls	C3	Serum	ELISA multiplex	Mean C3 not significantly different between cases (0.97 mg/ml) and controls (0.91 mg/ml)	Cases had first episode paranoid SZ
Spivak et al., 1989	Israel	Inpatient	DSM-III	Drug-free (≥3 months)	20 cases vs. 18 controls	C3, C4	Serum	Radial immunodiffusion	Mean C3 not significantly different between cases (146.1 mg/100 ml) vs. controls (136.2 mg/100 ml) Mean C4 not significantly different between cases (35.3 mg/100 ml) and controls (36.7 mg/100 ml)	Cases had chronic SZ Controls recruited from medical staff
Spivak et al., 1993	Israel	Inpatient	DSM-III-R	Drug-naïve, drug-free (≥3 months) and medicated	91 cases (20 drug-naïve, 37 drug-free, 34 medicated) vs. 37 controls	C3, C4	Serum	Radial immunodiffusion	Mean C3 not significantly different between cases (128 mg/100 ml) vs. controls (132 mg/100 ml) Mean C4 not significantly different between cases (33 mg/100 ml) and controls (35 mg/100 ml) No significant differences for C3 or C4 in cases vs. controls when stratified by medication status	Cases had chronic SZ Controls recruited from medical staff
Walss-Bass et al., 2019	USA	Outpatient	DSM-IV-TR	Medicated	60 cases vs. 20 controls	C4	Plasma	ELISA	Mean C4 significantly increased in cases (242.5 μg/ml) vs. controls (191.8 μg/ml), *p* = .0097	Cases had chronic SZ
Wong et al., 1996	Singapore	Inpatient	ICD-9	Medicated and drug-free (15 drug-free)	Series 1: 44 recovered (neither positive nor negative symptoms), 30 chronic (only negative symptoms), 15 acute (positive and negative symptoms) vs. 41 controls Series 2: 50 acute cases (positive and negative symptoms) vs. 50 controls	C3	Serum	Immuno-electrophoresis	Series 1: Mean C3 significantly ↓ in chronic cases (0.75 g/l) vs. controls (0.88 g/l), p < .01 No significant differences for acute or recovered patients compared to controls Series 2: mean C3 not significantly different between acute cases (0.81 g/l) vs. controls (0.70 g/l)	All participants were males Controls in series 1 were blood donors Controls in series 2 were healthy Chinese male civil servants attending compulsory medical examination No significant differences in C3 in cases on or off antipsychotics

SZ: schizophrenia; ELISA: Enzyme-linked immunosorbent assay; ICD: International Classification of Diseases; DSM: Diagnostic and Statistical Manual; MBL: mannose-binding lectin; MASP: mannose-binding lectin serine protease.

All included studies were of a case-control design and defined the diagnosis of schizophrenia in cases according to contemporaneous International Classification of Diseases (ICD) or Diagnostic and Statistical Manual (DSM) criteria. Sample sizes ranged from 34 to 450 participants. In the majority of included studies assays were performed using serum samples; five studies instead used plasma (Hong et al., 2016b; Kucharska-Mazur et al., 2014; Li et al., 2016; Maes et al., 1997; Walss-Bass et al., 2019). Thirteen studies recruited inpatient cases (Boyajyan et al., 2010; Fontana et al., 1980; Hakobyan et al., 2005; Hong et al., 2016b; Idonije et al., 2012; Ji et al., 2019; Li et al., 2016; Maes et al., 1997; Sasaki et al., 1994; Schwarz et al., 2012; Spivak et al., 1993; Spivak et al., 1989; Wong et al., 1996), seven studies recruited outpatients (Ali et al., 2017; Laskaris et al., 2018; Li et al., 2012; Mayilyan et al., 2006; Mayilyan et al., 2008a; Santos Soria et al., 2012; Walss-Bass et al., 2019), one recruited both inpatient and outpatient cases (Foldager et al., 2012) and in the remainder the hospitalisation status of patients was not determined (Cazzullo et al., 1998; Kucharska-Mazur et al., 2014; Ramsey et al., 2013). In many studies, the clinical status of patients at the time of blood sampling (i.e. in remission or acute psychosis) was unclear. In at least four studies (Cazzullo et al., 1998; Foldager et al., 2012; Hakobyan et al., 2005; Sasaki et al., 1994) sampling was performed when patients were experiencing acute exacerbations or active symptoms of schizophrenia, while two studies stated that cases were stable or in remission (Mayilyan et al., 2008a; Santos Soria et al., 2012). Most studies recruited cases with chronic schizophrenia, although in five studies all (Ji et al., 2019; Kucharska-Mazur et al., 2014; Ramsey et al., 2013; Schwarz et al., 2012) or a proportion (Idonije et al., 2012) of cases were in their first episode of illness. Most studies included participants who were prescribed antipsychotics at the time of sampling, of which five reported data stratified by medication status (Boyajyan et al., 2010; Hakobyan et al., 2005; Idonije et al., 2012; Maes et al., 1997; Spivak et al., 1993). In others, cases were drug-naïve (never exposed to antipsychotic therapy) (Hong et al., 2016b; Kucharska-Mazur et al., 2014; Ramsey et al., 2013; Schwarz et al., 2012) or drug-free for a period of time (Ali et al., 2017; H. Li et al., 2016; Y. Li et al., 2012; Spivak et al., 1989) although in the latter case, the duration of time off medication prior to blood sampling was variable (from one week to >3 months).

#### 3.2.1 Assays used

The included studies utilised several different methodologies to measure complement proteins. Seven studies used activity-based assays measuring haemolytic or functional activity of complement pathways or proteins (Table 1). Twenty studies quantified complement protein concentrations rather than activity (Table 2). Of these, ten used enzyme-linked immunoassay (ELISA), four used radial immunodiffusion, two used nephelometry and the remainder used other techniques.

#### 3.2.2 Risk of bias assessment

The median quality score according to the Newcastle-Ottawa scale was three and the range of scores was from one to seven (see Supplementary Appendix B).

### 3.3 Qualitative synthesis of results

#### 3.3.1 Activity-based assays

Of the seven studies assessing complement activity (Table 1), six studies measured activity of the classical pathway. One study found total complement activity to be significantly lower in cases compared to controls (Spivak et al., 1993), one found total complement activity significantly increased in cases (Mayilyan et al., 2006) and four found no evidence of a significant difference (Hakobyan et al., 2005; Li et al., 2012; Sasaki et al., 1994; Spivak et al., 1989).

Two studies measured alternative pathway activity, which was found to be significantly increased in medicated and non-medicated cases compared to controls in one study (Boyajyan et al., 2010) though significantly reduced in cases compared to controls in the other study (Li et al., 2012).

Activity of the MBL pathway was measured in one study (Li et al., 2012) which found no evidence of a significant difference between cases and controls.

Regarding activity of individual proteins, C3 activity was measured in two studies and found to be significantly increased relative to controls (Boyajyan et al., 2010; Hakobyan et al., 2005) as well as in patients on antipsychotic therapy compared to non-medicated patients (Hakobyan et al., 2005). C4 activity was measured in two studies, both of which found significantly increased activity compared to controls (Hakobyan et al., 2005; Mayilyan et al., 2006). C1 and C2 activity were measured in one study (Hakobyan et al., 2005) which found that C1 activity was significantly increased and C2 activity significantly decreased in cases compared to controls. One study found MBL-bound mannose-binding lectin serine peptidase 2 (MASP2) activity was significantly increased in cases relative to controls, although the same study reported no significant difference in MBL-bound MASP1 activity (Mayilyan et al., 2006).

#### 3.3.2 Concentration-based assays

Twenty studies measured serum or plasma concentrations of specific complement proteins in schizophrenia patients and controls (Table 2). In total, concentrations of 13 different complement proteins were assessed across all included studies.

Twelve studies reported peripheral concentrations of C3, of which three studies found significantly increased levels in serum from cases compared to controls (Ali et al., 2017; Ramsey et al., 2013; Santos Soria et al., 2012). One study found significantly decreased levels in cases (Li et al., 2016). Another study reported a similar finding in one of two case-control series, but this was not replicated in the other series within the same study (Wong et al., 1996). The remaining studies reported no evidence of a significant difference between cases and controls (Cazzullo et al., 1998; Fontana et al., 1980; Hong et al., 2016b; Laskaris et al., 2018; Schwarz et al., 2012; Spivak et al., 1993; Spivak et al., 1989).

Ten studies reported C4 concentrations, of which two found significantly increased levels in cases (Laskaris et al., 2018; Walss-Bass et al., 2019), one found significantly decreased levels in cases (Ji et al., 2019) and seven studies found no significant difference (Ali et al., 2017; Fontana et al., 1980; Idonije et al., 2012; Maes et al., 1997; Santos Soria et al., 2012; Spivak et al., 1993; Spivak et al., 1989). One of these studies stratified cases by medication status (Maes et al., 1997) and found increased serum C4 in drug-free patients compared to controls without schizophrenia (Maes et al., 1997). However, the minimal specified duration off antipsychotics was short (at least one week) and the sample size was small (ten medicated and 17 drug-free patients).

Two studies reported C3c concentrations. One study (Idonije et al., 2012) found no significant difference between cases and controls. The other (Maes et al., 1997) reported no difference between medicated schizophrenia patients and controls, despite evidence of a significant increase in drug-free patients compared to controls. However, the same caveats apply as described for the same study in relation to C4.

Two studies reported C1 inhibitor concentrations, both of which found no evidence of a significant difference between cases and controls (Fontana et al., 1980; Idonije et al., 2012). Two studies reported C1q concentrations. In one study, concentration of C1q was significantly lower in both medicated and antipsychotic-free cases compared to controls (Idonije et al., 2012) although no difference was found in the other study measuring this protein (Laskaris et al., 2018).

Two studies reported concentrations of MBL (Foldager et al., 2012; Mayilyan et al., 2006). In both studies, no significant difference was found on primary analysis between cases and controls. However, in one study (Foldager et al., 2012), a significant increase in cases was reported following adjustment for MBL2 haplotype.

Concentrations of C4b, C5, C3 activator, MASP2, C3a, C5a and C5b-9 were measured in individual studies. C4b was found to be significantly reduced in cases (Mayilyan et al., 2008a). C5 was unchanged between cases and controls but was increased in chronically unwell medicated patients compared to newly-diagnosed drug-free patients (Idonije et al., 2012). For both C3 activator (Idonije et al., 2012) and MASP2 (Foldager et al., 2012) no significant differences were reported. In a study measuring C3a, C5a and C5b-9 in patients with first-episode schizophrenia, C3a was significantly reduced in cases compared to controls, but no significant differences were reported for C5a or C5b-9 (Kucharska-Mazur et al., 2014).

### 3.4 Meta-analyses

#### 3.4.1 Complement component 3

For C3, sufficient data were available to perform meta-analysis of 11 studies (Ali et al., 2017; Fontana et al., 1980; Hong et al., 2016b; Laskaris et al., 2018; Li et al., 2016; Santos Soria et al., 2012; Schwarz et al., 2012; Spivak et al., 1993; Spivak et al., 1989; Wong et al., 1996). One study (Wong et al., 1996) presented data for two different case-control series which were considered as separate studies in the meta-analysis. In one of these series, data for cases were presented stratified by symptomatology into three groups (positive and negative symptoms; only negative symptoms; no symptoms) which were compared separately to the same group of healthy controls. To facilitate comparison with the other studies, we calculated the pooled mean and standard deviation across the three groups of cases and used these values in the meta-analysis.

**Fig. 2:**
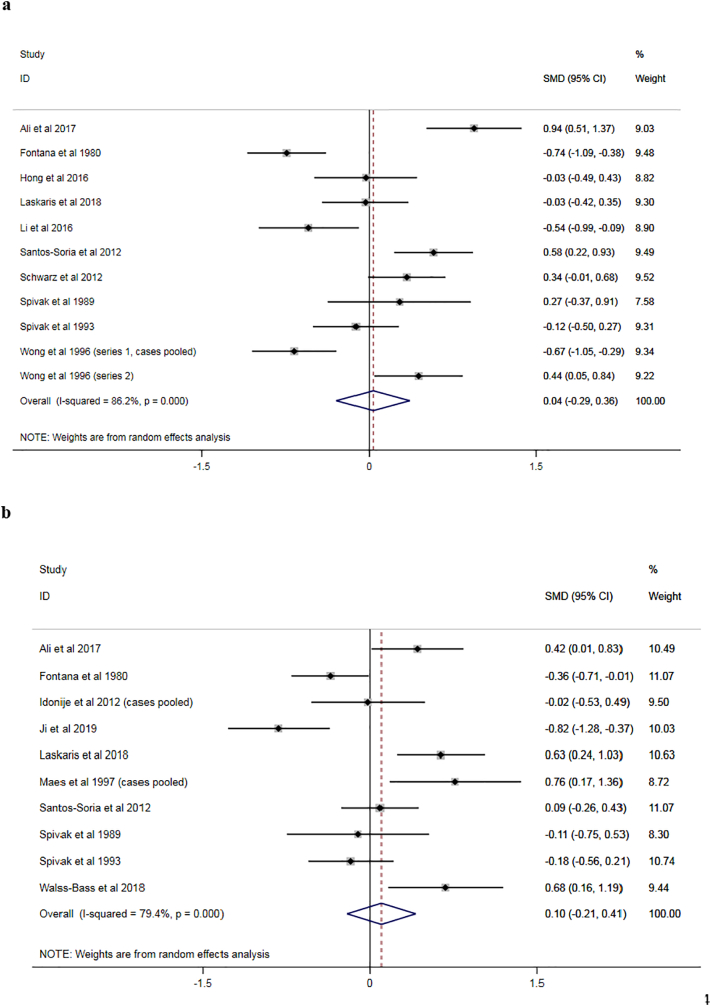
**a:** Forest plot for meta-analysis of studies measuring peripheral complement component 3 concentration. **b:** Forest plot for meta-analysis of studies measuring peripheral complement component 4 concentration. SMD: standardised mean difference; 95% CI: 95% confidence interval.

Results of the meta-analysis are presented in the forest plot in Fig. 2a. The pooled effect estimate showed no evidence for a significant difference between cases and controls (SMD 0.04, 95% confidence interval [CI] −0.29–0.36; Fig. 2a). There was evidence of significant between-study statistical heterogeneity (*I*^2^ 86.2%, *p* < 0.001).

#### 3.4.2 Complement component 4

For C4, sufficient data were available to perform meta-analysis of ten studies (Ali et al., 2017; Fontana et al., 1980; Idonije et al., 2012; Ji et al., 2019; Laskaris et al., 2018; Maes et al., 1997; Santos Soria et al., 2012; Spivak et al., 1993; Spivak et al., 1989; Walss-Bass et al., 2019). In one study (Idonije et al., 2012) data for cases were presented stratified by stage of illness (chronic vs. FEP) and the two groups were compared separately to the same group of healthy controls. Another study (Maes et al., 1997) presented data for cases stratified by medication status (drug-free vs. on antipsychotics) and the two groups were compared separately to the same group of healthy controls. In both cases, we calculated the pooled means and standard deviations across groups of cases and used these values in the meta-analysis.

Results of the meta-analysis are presented in the forest plot in Fig. 2b. The pooled effect estimate showed no evidence for a significant difference between cases and controls (SMD 0.10, 95% CI −0.21–0.41). There was evidence of significant between-study statistical heterogeneity (*I*^2^ 79.4%, *p* < 0.001).

#### 3.4.3 Publication bias

For both C3 and C4, inspection of funnel plots did not indicate asymmetry suggestive of significant publication bias and Egger's test for small-study effects was not significant (see supplementary appendix C).

#### 3.4.4 Meta-regression analyses

For both C3 and C4, none of the characteristics examined (antipsychotic use, hospitalisation status and assay methodology) showed evidence of significant effects upon the pooled estimates (see supplementary appendix D).

## 4 Discussion

To our knowledge, this is the first systematic literature review and meta-analysis of serological studies measuring complement system activity or protein concentrations in patients with schizophrenia compared to controls. Among studies measuring activity of the classical pathway, activity did not significantly differ between cases and controls in four of six studies. Conflicting results were noted in two studies of alternative pathway activity. Individual studies measuring the classical and MBL pathways reported no evidence of a significant difference. Among studies measuring specific complement protein concentrations, C3 and C4 were the most frequently examined proteins. In meta-analyses, we found no evidence of significant differences between cases and controls for C3 or C4. Regarding other proteins, C1 inhibitor concentration did not differ in two studies. C1q concentration was reduced in one study but did not differ in a second study. No difference was found in two studies of MBL levels. For proteins measured in individual studies, a significant decrease was noted for C4b and C3a, but no significant changes were described for C3 activator, C5, C5a, C5b-9 or MASP2.

Collectively, evidence from these serological studies presents a mixed picture regarding changes in key peripheral complement proteins in schizophrenia. Whereas individual studies report significant differences in activity or concentrations of certain proteins in cases compared to controls, there is little evidence of a consistent pattern of change across studies. However, for most proteins, the available evidence is scarce, limiting our ability to draw broad inferences. Furthermore, the available studies are methodologically and clinically diverse.

### 4.1 Methodological diversity

Regarding methods of measurement, the majority of studies examining activity of the complement system used haemolytic activity assays. However, studies that quantified complement protein concentrations used a range of methods with varying degrees of sensitivity (Bouzek and Mancal, 1989; Koelle and Bartholomew, 1982; Mali et al., 2009), limiting comparisons between studies. Effect sizes for individual proteins may be small and potentially undetectable by relatively insensitive methods. Differences with respect to sample medium, handling, processing and storage between studies are also relevant. For example, thawing effects are exaggerated for some biomarkers of complement activation in serum and citrated plasma, but not in plasma treated with ethylenediaminetetraacetic acid (Yang et al., 2015). It is also possible that specific isoforms of certain complement proteins are changed or dysregulated in cases relative to controls, but outside of mass spectrometric methods there is limited availability of assays that accurately differentiate between multiple isoforms in this way.

Differences in study design may further contribute to heterogeneity in results. For example, some studies introduced bias by recruiting controls from medical staff or other potentially non-representative sources such as blood donors. Many studies did not adequately control for potential confounders such as age (Stephan et al., 2013), body mass index (Cominetti et al., 2018; Yang et al., 2003) or disorders of physical health such as diabetes mellitus (Engstrom et al., 2005). Finally, in most studies the sample size was small, and possibly under-powered to detect meaningful differences for multiple proteins between cases and controls.

### 4.2 Clinical diversity

Differences related to clinical characteristics of the patient samples in the included studies also limit generalisable inferences. For example, studies differed on whether patients were never, previously or currently prescribed antipsychotic medication. The effects of antipsychotic medication on the complement system have not been widely investigated. However, antipsychotics have a range of immunomodulatory effects including acting upon cytokine networks (Drzyzga et al., 2006; Pollmacher et al., 2000) and through these may exert influence on the complement system. Several of the included studies presented results stratified by medication status, with conflicting results. Among studies assessing complement activity, Boyajyan et al. (2010) found increased alternative pathway and C3 activity in medicated and drug-free patients compared to controls. Hakobyan et al. (2005) found increased C3 activity in medicated compared to non-medicated patients. Li et al. (2012) found decreased alternative pathway activity in drug-free compared to medicated patients. Spivak et al. (1993) reported no difference in total complement activity between medicated or non-medicated cases and controls. Among studies measuring complement protein concentrations, Maes et al. (1997) reported increased C3c and C4 in drug-free patients versus controls, but not in patients currently taking antipsychotics. Spivak et al. (1993) reported no difference in medicated compared to non-medicated cases with respect to C3 and C4 concentrations. Idonije et al. (2012) compared controls with two groups of cases: patients with chronic schizophrenia who were prescribed antipsychotics, and patients with first-episode schizophrenia who were drug-free. As such, it is not possible when interpreting the results from this study to separate the effects of stage of illness and medication. Nevertheless, C1q was found to be reduced in both sets of patients compared to controls; C3c was reduced only in the first-episode drug-free group; and no differences relative to controls were found for C4, C5, C1 inhibitor or C3 activator. To summarise, there is some limited evidence that antipsychotic medication may influence the complement system, but the precise nature of these effects requires further study.

With regard to stage of illness, relatively few studies have investigated complement proteins in patients at different stages of disorder within the same study. There is however value in investigating biomarkers in patients at different stages of illness, and especially in early stages, to gain aetiopathogenic insights and, potentially, to inform early intervention strategies. One included study (Laskaris et al., 2018) compared C1q, C3 and C4 levels in healthy controls with three groups of cases: patients with chronic schizophrenia, patients with FEP and individuals at clinical high-risk (CHR) for psychosis (Yung et al., 2005). Compared to controls, C1q levels did not differ for each of the three groups of cases, C3 was higher in CHR and C4 was higher in CHR and chronic schizophrenia. The sample size for the CHR group was small (ten participants), but these results preliminarily suggest that complement protein changes may be present in early psychosis phenotypes.

### 4.3 Evidence in context

There have been mixed findings regarding complement polymorphisms in candidate gene studies as reviewed previously by Mayilyan et al. (2008b) and more recently by Woo et al. (2019), although genome-wide association studies in schizophrenia provide strong evidence for implication of the major histocompatibility complex (Consortium, S. W. G. o. t. P. G, 2014; Purcell et al., 2009; Ripke et al., 2013). This association was later partly explained by allelic variation of C4 (Sekar et al., 2016). C4 RNA expression was found to be increased in post-mortem brain samples from patients with schizophrenia compared to controls, and on immunohistochemical analysis was noted to localise to neurons and synapses (Sekar et al., 2016). In concert with previous data (Stevens et al., 2007), these findings suggest a role for complement in contributing to synaptic pruning. This process is thought to be excessive in schizophrenia, given evidence of reduced synaptic density in association with the disorder (Glausier and Lewis, 2013; Osimo et al., 2018). Complement proteins may serve other functions in the central nervous system such as influencing neuronal migration in neurodevelopment (Gorelik et al., 2017) and neural plasticity after ischaemic injury (Stokowska et al., 2017). Most complement proteins do not cross the blood-brain barrier, and thus it is possible that concentrations of complement proteins in serum or plasma do not reflect complement activity in the brain. Indeed, this may also in part explain the lack of consensus identified from serological studies in schizophrenia. However, the complement system and blood-brain barrier interact with one another (Alexander, 2018; Jacob et al., 2010) and this relationship might be especially relevant in psychosis, given evidence of associated blood-brain barrier dysfunction (Pollak et al., 2018) that might facilitate passage of peripherally-derived complement proteins into the central nervous system, or vice-versa.

Notwithstanding the methodological and clinical diversity between studies, the findings of this review may be in keeping with the hypothesis that significant immune dysregulation occurs in only a sub-population of patients with schizophrenia (Miller and Goldsmith, 2017; Schwarz et al., 2014). In potential support of this view, the high statistical heterogeneity in our meta-analyses of C3 and C4 was not fully explained by between-study variation in antipsychotic use, hospitalisation status or assay methodology. Some of this heterogeneity may reflect biological differences among patients diagnosed with schizophrenia. Higher baseline levels of pro-inflammatory cytokines predict treatment non-response in FEP (Mondelli et al., 2015) suggesting that patients with an ‘inflammatory subtype’ may be more likely to have more severe illness. Such patients may be less likely to respond optimally to usual treatments and may potentially benefit from adjunctive anti-inflammatory or immunomodulatory agents (Cho et al., 2019). Whether there are one or several such immunophenotypes in schizophrenia, potentially characterised by differences in the precise nature and extent of immune disturbances, remains to be determined (Miller and Goldsmith, 2017).

It may also be the case that significant complement dysregulation occurs early in the course of disorder but becomes quiescent by the time of schizophrenia diagnosis (potentially several years after the initial onset of psychosis), and thus is not detectable relative to controls in cross-sectional studies. In support, a recent study in the earlier and broader phenotype of FEP found higher levels of the membrane attack complex in patients compared to controls (Kopczynska et al., 2017). Further evidence that certain complement changes occur early in the psychosis spectrum comes from mass spectrometry-based proteomic studies in non-clinical general population samples showing differential expression of complement proteins in age 12 plasma samples from individuals who go on to report psychotic experiences at age 18 compared to controls who do not (English et al., 2018; Föcking et al., 2019). Another prospective study found that maternal immunoglobulin G markers of C1q were higher in mothers whose children later developed schizophrenic or affective psychosis compared to mothers whose children did not develop mental disorder (Severance et al., 2014) suggesting that these disturbances may even span generations.

Dysregulation of the complement system may also be associated with phenotypes outside of the schizophrenia or psychosis spectrum. Complement protein changes have been observed in other mental disorders (Comes et al., 2018; Druart and Le Magueresse, 2019) including depression (Chen et al., 2015; Stelzhammer et al., 2014), bipolar disorder (Reginia et al., 2018) and autism spectrum disorder (Corbett et al., 2007; Fagan et al., 2017). Thus these changes may reflect shared molecular pathology and aetiological mechanisms between these clinical phenotypes (Druart and Le Magueresse, 2019). The possible primary and perpetuating causes of complement dysregulation and inflammation in schizophrenia and other mental disorders are unclear, but maternal immune activation (Conway and Brown, 2019), exposure to infections (Kneeland and Fatemi, 2013), the microbiome (Severance and Yolken, 2019) and other environmental factors such as childhood adversity (Danese and J Lewis, 2017) and substance use (Miller et al., 2018) are potential contributing factors. Drugs targeting the complement system are available and others in active development (Carpanini et al., 2019; Druart and Le Magueresse, 2019; Ricklin et al., 2018; Zelek et al., 2019), but whether they may prove useful in the treatment or prevention of psychotic disorders will require extensive preclinical testing before human trials.

### 4.4 Limitations

At the study level, as previously discussed, most studies included in this review were of small sample size and potentially inadequately powered. Studies were generally prone to risk of bias, and reporting of data was inconsistent. In particular, in many studies it was unclear whether the case definition was independently validated; few studies reported the non-response rate in terms of recruitment of cases and controls; and several studies did not adequately control for possible confounders (such as BMI, tobacco and illicit drug use) in the design or analysis.

At the review level, some limitations should be noted. Firstly, our search strategy was limited to studies published in English. Secondly, our focus was on targeted methods of complement protein measurement and thus this synthesis does not include results from proteome-wide investigations. Thirdly, we only included studies published in peer-reviewed academic journals, potentially at the expense of including an increased breadth of literature as derived from grey literature sources such as posters and conference abstracts.

### 4.5 Conclusions

The available evidence from serological studies is inconsistent regarding dysregulation of the complement system in schizophrenia. Studies have generally been small with methodological and clinical heterogeneity. Further studies in larger samples would help to clarify the potential role of complement proteins as biomarkers. Recent evidence from genetic and proteomic studies has suggested that complement changes are detectable and may occur early in the course of disorder. Large-scale prospective studies with repeated biosampling would be helpful to examine longitudinal changes in complement proteins in early and later development of psychotic and other mental disorders. Studies have begun to focus on measurement of complement proteins in early phenotypes, such as patients in their first episode of psychosis and in clinical high-risk individuals. Pursuing these promising lines of investigation will provide illuminating insights regarding the aetiopathogenic role of the complement system and potential attendant opportunities for early intervention.

## Contributions

DM, SRS, MF and DRC contributed to the conception and design of the review. DM and SS performed the database search and data extraction. DM performed the statistical analyses. DM, SS, SRS, MF, MC and DRC contributed to the drafting and review of the manuscript.

## Role of the funding source

DM is a Fellow on the Irish Clinical Academic Training (ICAT) Programme, which is supported by the 10.13039/100010269Wellcome Trust and the 10.13039/100010414Health Research Board (Grant Number 203930/B/16/Z), the Health Service Executive National Doctors Training and Planning and the Health and Social Care, Research and Development Division, Northern Ireland. MC was supported by a 10.13039/501100000781European Research Council Consolidator Award (iHEAR 724809). The funders had no involvement in study design; in the collection, analysis and interpretation of data; in the writing of the report; and in the decision to submit the article for publication.

## Declaration of competing interest

All authors declare no conflicts of interest in relation to this work.

## Appendix A Supplementary data

Appendix A Search terms used for each database. Appendix B Risk of bias assessment. Appendix C Assessment of publication bias and small-study effects. Appendix D Results of meta-regression analyses. Image 1
