# Supplementary material for: Peripheral complement proteins in schizophrenia: A systematic review and meta-analysis of serological studies
Source: Schizophr Res. 2020 Aug;222:58–72. doi: 10.1016/j.schres.2020.05.036 (PMC7594643; doi:10.1016/j.schres.2020.05.036)
Supplement: Supplementary file 1 — Appendix A Search terms used for each database. Appendix B Risk of bias assessment. Appendix C Assessment of publication bias and small-study effects. Appendix D Results of meta-regression analyses. [file mmc1.pdf]

SUPPLEMENTARY APPENDICES

Appendix A: Search terms used for each database

Appendix B: Risk of bias assessment based on the Newcastle Ottawa scale for case control studies

Appendix C: Assessment of publication bias and small-study effects

Appendix D: Results of meta-regression analyses

Appendix A: Search terms used for each database

PubMed<br>Medline	
**1**	(("Schizophrenia"[Mesh]) OR "Psychotic Disorders"[Mesh]) OR ((((schizophrenia[Title/Abstract]) OR schizophrenic[Title/Abstract]) OR psychosis[Title/Abstract]) OR psychotic[Title/Abstract])
**2**	("Complement System Proteins"[Mesh]) OR complement[Title/Abstract]
**3**	**1 AND 2**

EMBASE<br>Elsevier	
**1**	'schizophrenia'/exp OR schizoprenia:ti,ab OR schizophrenic:ti,ab OR 'psychosis'/exp OR psychosis:ti,ab OR psychotic:ti,ab
**2**	'complement'/exp OR complement:ti,ab,de
**3**	1 AND 2
**4**	**#3 AND [embase]/lim NOT ([embase]/lim AND [medline]/lim)**<br><br>**LIMITED TO EMBASE ONLY RECORDS, EXCLUDING MEDLINE**

PSYCINFO<br>Ebscohost	
1	DE "Schizophrenia" OR DE "Acute Schizophrenia" OR DE "Catatonic Schizophrenia" OR DE "Childhood Schizophrenia" OR DE "Paranoid Schizophrenia" OR DE "Process Schizophrenia" OR DE "Schizophrenia (Disorganized Type)" OR DE "Schizophreniform Disorder" OR DE "Undifferentiated Schizophrenia"
2	DE "Psychosis" OR DE "Acute Psychosis" OR DE "Affective Psychosis" OR DE "Alcoholic Psychosis" OR DE "Capgras Syndrome" OR DE "Childhood Psychosis" OR DE "Chronic Psychosis" OR DE "Experimental Psychosis" OR DE "Hallucinosis" OR DE "Paranoia (Psychosis)" OR DE "Postpartum Psychosis" OR DE "Reactive Psychosis" OR DE "Schizophrenia" OR DE "Senile Psychosis" OR DE "Toxic Psychoses"
3	(TI schizophrenia OR schizophrenic OR psychosis OR psychotic) OR (AB schizophrenia OR schizophrenic OR psychosis OR psychotic)
4	**1 OR 2 OR 3**
5	(TI complement) OR (AB complement)
6	**4 AND 5**

Appendix B: Risk of bias assessment based on the Newcastle-Ottawa scale for case-control studies

Study	Selection score (maximum 4)	Comparability score (maximum 2)	Exposure score (maximum 3)	Total score (maximum 9)
**Ali et al 2017**	3	2	2	7
**Boyajyan et al 2010**	2	0	0	2
**Cazzullo et al 1998**	1	1	0	2
**Foldager et al 2012**	0	1	0	1
**Fontana et al 1980**	1	0	0	1
**Hakobyan et al 2004**	2	0	0	2
**Hong et al 2016**	2	2	0	4
**Idonije et al 2012**	1	2	0	3
**Ji et al 2019**	3	0	0	3
**Kucharska-Mazur et al 2014**	2	2	0	4
**Laskaris et al 2018**	1	2	1	4
**Li et al 2012**	2	1	0	3
**Li et al 2016**	1	2	1	4
**Maes et al 1997**	1	2	0	3
**Mayilyan et al 2006**	0	2	0	2
**Mayiliyan et al 2008**	3	1	0	4
**Ramsey et al 2013**	2	2	1	5
**Santos-Soria et al 2012**	1	2	0	3
**Sasaki et al 1994**	1	0	0	1
**Schwarz et al 2012**	3	2	0	5
**Spivak et al 1989**	0	1	0	1
**Spivak et al 1993**	1	1	0	2
**Walls-Bass et al 2018**	2	2	1	5
**Wong et al 1996**	0	1	0	1

Wells G, Shea B, O'Connell D, Peterson J, Welch V, Losos M, et al. The Newcastle-Ottawa Scale (NOS) for assessing the quality of nonrandomised studies in meta-analyses. Available from: http://www.ohri.ca/programs/clinical_epidemiology/oxford.asp.

Appendix C: Assessment of publication bias and small-study effects

1) Funnel plot and Egger's test for complement component 3

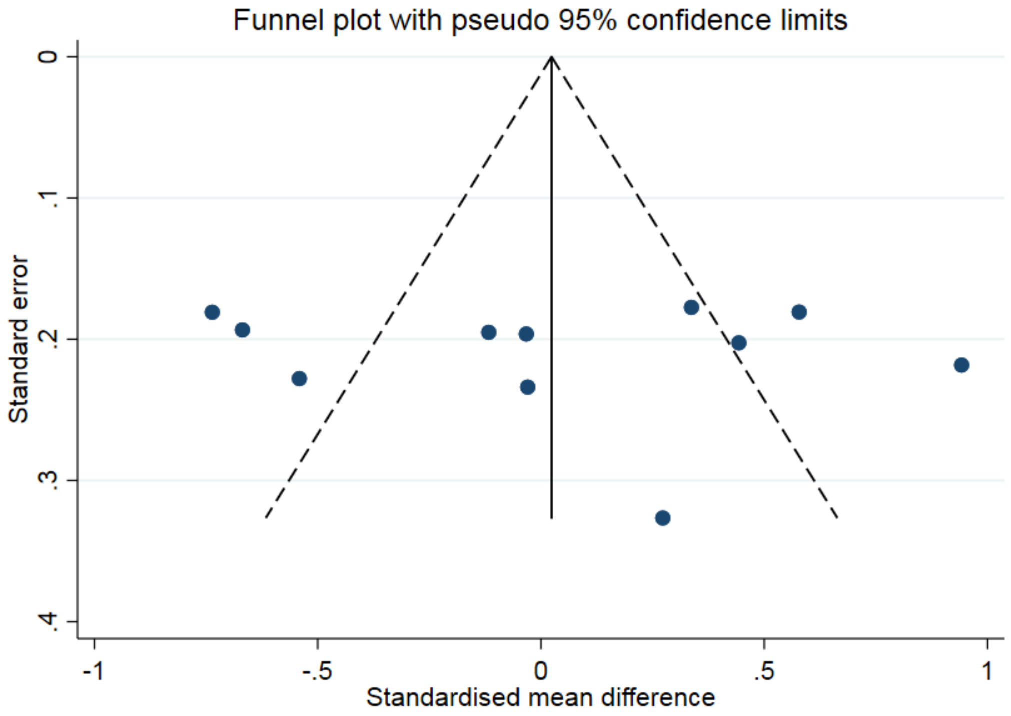

Egger's test for small-study effects:

t=0.28, *p*=0.789

2) Funnel plot and Egger's test for complement component 4

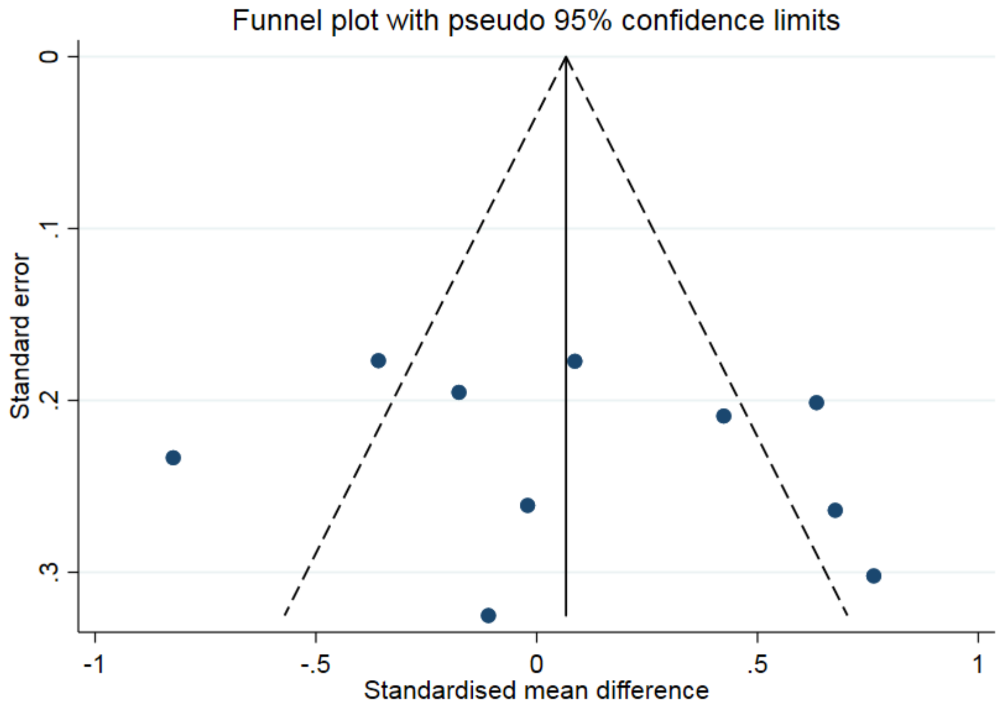

Egger's test for small-study effects:

t=0.67, *p*=0.524

Appendix D: Results of meta-regression analyses

1) Meta-regression analyses for complement component 3

Characteristic	Number of studies	Coefficient	Standard error	t	*p*	Lower 95% confidence interval	Upper 95% confidence interval
Current antipsychotic use (yes vs. no)	11	-0.29	0.33	-0.86	0.412	-1.05	0.47
Hospitalisation status (inpatient vs. outpatient)	11	-0.63	0.32	-1.96	0.082	-1.36	0.10
Assay (ELISA vs radial immunodiffusion/other)	11	-0.15	0.36	-0.43	0.679	-0.96	0.65
Assay (radial immunodiffusion vs ELISA/other)	11	-0.36	0.37	-0.97	0.357	-1.20	0.48
Assay (other vs ELISA/ radial immunodiffusion)	11	0.45	0.33	1.38	0.200	-0.29	1.19

2) Meta-regression analyses for complement component 4

Characteristic	Number of studies	Coefficient	Standard error	t	*p*	Lower 95% confidence interval	Upper 95% confidence interval
Current antipsychotic use (yes vs. no) [a]	9	0.01	0.37	0.02	0.988	-0.86	0.87
Hospitalisation status (inpatient vs. outpatient)	10	-0.60	0.27	-2.20	0.059	-1.22	0.03
Assay (ELISA vs radial immunodiffusion/other)	10	0.09	0.37	0.23	0.821	-0.77	0.94
Assay (radial immunodiffusion vs ELISA/other)	10	-0.31	0.42	-0.73	0.488	-1.28	0.66
Assay (other vs ELISA/ radial immunodiffusion)	10	0.12	0.34	0.34	0.740	-0.67	0.90

[a] Medication status was unable to be confirmed for one study (Ji et al 2019) and thus this study was not included in this specific analysis.

ELISA: enzyme-linked immunosorbent assay
